# Predicting synchronized gene coexpression patterns from fibration symmetries in gene regulatory networks in bacteria

**DOI:** 10.1186/s12859-021-04213-5

**Published:** 2021-07-08

**Authors:** Ian Leifer, Mishael Sánchez-Pérez, Cecilia Ishida, Hernán A. Makse

**Affiliations:** 1 Levich Institute,Physics Department, City College of New York, New York, NY 10031 USA; 2 Programa de Genómica Computacional, Centro de Ciencias Genómicas, Universidad Nacional Autónoma de México, Cuernavaca, Mexico; 3 Faculty of Medicine and Biomedical Sciences, Autonomous University of Chihuahua, 31125 Chihuahua, Chihuahua Mexico

## Abstract

**Background:**

Gene regulatory networks coordinate the expression of genes across physiological states and ensure a synchronized expression of genes in cellular subsystems, critical for the coherent functioning of cells. Here we address the question whether it is possible to predict gene synchronization from network structure alone. We have recently shown that synchronized gene expression can be predicted from symmetries in the gene regulatory networks described by the concept of symmetry fibrations. We showed that symmetry fibrations partition the genes into groups called fibers based on the symmetries of their ’input trees’, the set of paths in the network through which signals can reach a gene. In idealized dynamic gene expression models, all genes in a fiber are perfectly synchronized, while less idealized models—with gene input functions differencing between genes—predict symmetry breaking and desynchronization.

**Results:**

To study the functional role of gene fibers and to test whether some of the fiber-induced coexpression remains in reality, we analyze gene fibrations for the gene regulatory networks of *E. coli* and *B. subtilis* and confront them with expression data. We find approximate gene coexpression patterns consistent with symmetry fibrations with idealized gene expression dynamics. This shows that network structure alone provides useful information about gene synchronization, and suggest that gene input functions within fibers may be further streamlined by evolutionary pressures to realize a coexpression of genes.

**Conclusions:**

Thus, gene fibrations provide a sound conceptual tool to describe tunable coexpression induced by network topology and shaped by mechanistic details of gene expression.

## Introduction

Gene regulation in bacteria has been studied since the time of the operon model of Jacob and Monod [1]. Knowing the regulators and mechanistic details of genes expression has greatly improved our understanding of cellular signal processing [2], and has lead to various applications in systems and synthetic biology [2–5]. General factors like RNA polymerase activity can lead to an overall increase or decrease of bacterial gene expression depending on cellular growth rate. The expression profiles of single genes are further regulated by specific transcription factors (TFs). At the same time, high-throughput expression studies have revealed the functional role of expression profiles: a variety of multivariate methods (including clustering, biclustering, plaid models, singular value decomposition, and Independent Component Analysis) have been used to extract functional information from expression profiles, often taking co-expression as a sign for shared biological function [2].

Gene regulation by transcription factors is described by gene regulatory networks (GRN) [5–7] where nodes are genes and a directed edge from gene A to gene B states that gene product of A is a TF that regulates the expression of B as an activator, repressor, or dual regulator. TF activities may be further modulated by signaling molecules that bind to the TF to activate or inactivate the protein. This process conveys information about the state of the cell, implementing for example a negative feedback control from metabolic synthesis pathways. In the gene regulatory network, such effector signaling molecules appear as external inputs to the GRN. The topologies of GRNs have been studied in detail [6] and been used as blueprints for dynamic models of gene expression [5]. Such models describe the production and degradation of gene products (mRNA or proteins) and consider the regulatory input functions of individual genes, which reflect TF binding to genes’ promoter regions, with gene-dependent binding parameters and possible binding states.

In principle, synchronized activity in gene expression [8–14] in bacteria reflects the gene arrangement in operons since genes in operons are transcribed together into a single mRNA molecule. A transcriptional unit (TU) is a set of contiguous genes that are transcribed into one mRNA. An operon is a set of contiguous genes controlled by the same promoter. However, beyond this trivial synchronization in gene expression, the largest part of the co-expression synchronization of gene activity can actually be attributed to specific pathways in the GRN regulated by specific TFs.

Quantitative gene expression models based on realistic input functions for all of the genes are out of reach due to the multiplicity of parameters defining these input functions [15]. Thus, there have been attempts to understand dynamic properties from network structure alone [15–18]. To establish GRNs for major model organisms, TF binding has been predicted from binding site sequences and based on ChIP/chip, ChIP-seq or ChIP-exo experiments. In these networks, motifs like the feed-forward loop [15, 17, 19] have been found by statistical methods, based on the frequent occurrence in networks, and have been studied as local signal processing circuits embedded in larger networks.

In a series of recent papers, we showed that symmetry principles applied to biology networks [20–22] can explain the synchronization in gene coexpression profiles. Synchronization here denotes joint evolution in time. That is, if each gene *i* in the network is represented by it’s expression level $x_i(t)$, as measured by the protein gene product with the time-scale approximations made, typically the concentration of mRNA in the cell, then genes *i* and *j* with expression levels $x_i$ and $x_j$ are synchronized if $lim_{t \rightarrow \infty }(x_i(t)-x_j(t)) \rightarrow 0$. We have found structural symmetries in biological networks, described by the concept of symmetry fibrations [20], that provide a principled way to define building blocks of genetic networks supporting synchronized gene expression. Symmetry fibrations are a powerful tool to describe the structure of networks that combines geometry and algebra data via category theory [23–25].

In this paper we first review the concept of symmetry fibration applied to GRNs in bacteria and we further address the question of gene synchronization and coexpression by comparing to existing experimental data, and ask how gene coexpression can be implemented by network structure. The methodology allows the user to find groups of genes that are active at the same time. These groups may define trap spaces (groups of related attractors, with their corresponding basins of attraction) or even specific attractors especially if the pattern of synchronous genes includes inactive genes as well as active ones. In this paper we address the question of how the behavior of the system relates to the synchronous gene groups. In particular, we challenge the predominant view that *coexpression* (by which two genes show similar expression profiles) is necessarily a sign of *coregulation* (by which these genes are controlled by a common transcription factor) [2, 26–39]. Instead, we claim that other, more complex circuits in the regulatory network can lead to coexpression. These circuits are identified by their symmetry properties and show synchronization in gene coexpression as a result of the underlying symmetries in the gene regulatory network.

In the case of GRNs, fibration symmetries can reveal meaningful building blocks in regulatory networks [20, 22]. In contrast to network motifs, which can only be found by their frequent occurrence, gene fibrations can find such circuits based on their symmetry properties even if these circuits are large and appear only once in the network. Furthermore, fibrations are mathematically proven to induce synchronization, which will be discussed in more detail in Chapter 2. The symmetry fibration is a transformation that reduces the networks to its base by collapsing symmetric genes into fibers. As will be discussed further, genes that belong to one fiber can show synchronous expression activity, resulting in coexpression patterns of all genes in the fiber. These circuits can perform signal processing tasks, namely generating structurally encoded, yet tunable patterns of gene coexpression. Other types of building blocks, such as densely connected modules defined by counting a ’density’ of edges within that module [40], are not expected to show this property.

Thus, our previous work [20, 22] shows that symmetry fibrations can group genes into fibers that point us to the synchronized building blocks of the GRN. Using the same approach, we managed to detect regulatory structures in the neural network of *C. elegans* worm and to give them a functional explanation [21]. If fibers can explain synchronization between cells, we can expect to see the same principle within cells, in the coexpression of genes and the underlying gene regulatory networks. Thus, as a working hypothesis, we pose that coexpression, arising from transcriptional regulation, can be directly understood through genome organization, gene conservation across genomes, and network structure, namely through gene regulatory building blocks related to fibers, while quantitative details of gene regulation play a minor role.

To treat networks as “blueprints” of dynamic gene regulation models, gene regulatory input functions must be defined. With simple identical input functions, and disregarding all quantitative differences (e.g. in binding parameters or mRNA half lives defining the input functions), genes in a fiber are predicted to show identical expression profiles. Under this hypothesis, fibration symmetries lead to important consequences for the dynamics of the gene expression in the network: fibrations give rise to the existence of synchronous solutions. In reality, however, input functions will differ between genes, and additional modulation of transcription factor activities by signaling molecules will come into play. The predicted symmetry will be broken and we expect a partial loss of synchronization. Genes *i* and *j* with dynamical solutions $x_i(t)$ and $x_j(t)$ that reach the state in which, after the time $t_{sync}$, solutions are $\varepsilon$-close are said to be “almost synchronized”. That is, two genes are almost synchronized if $|x_i(t)-x_j(t)| < \varepsilon$ for $t \in [t_{sync}, \infty ]$. It was shown [41] that slight mismatch in the parameters leads to solutions with nearly synchronous trajectories, that is, almost synchronized solutions. Detailed analysis of the situation with the difference in the input functions hasn’t been done in the literature so far, but basing on the conclusion in the networks with slight mismatch, we assume that in the networks with “slightly bigger” mismatch perfect synchronization will be broken even further. We hypothesize that precise coexpression, as predicted by our idealized model, will be reflected in cells in a partial coexpression that may be tuned by external biochemical signals.

Each gene (node of the network) and it’s time evolution is thought of as one variable—it’s expression level. Thus, it may look like this method is not applicable to more complex organisms because processes like transcription, translation, folding and binding to DNA are lumped into one step and sophisticated effects like RNA splicing, DNA methylation, histone acetylation, and overwinding or underwinding of DNA are ignored. However, fibration theory can still be applied even when one node has a very complicated behavior that takes all of the above into consideration. For example, we can extend each node to two variables—mRNA and protein concentration, and then the dynamics of the symmetric nodes will be synchronous by variable. That is, in this example synchronous nodes will have both equal mRNA and protein concentrations. As discussed above, each level of complexity has a potential to create more symmetry breaking. Thus, whether gene fibrations are useful in practice depends on how much of the coexpression remains in reality. In this paper we study this question in detail by studying coexpression in *Escherichia coli* and *Bacillus subtilis*, the major Gram-positive and Gram-negative bacterial model organisms: we predict a set of coexpressed genes based on fibration symmetry and confront these predictions with transcriptome data. The GRNs under study, which we analysed before in [20, 22], contain various types of fibers, including feed-forward fibers, Fibonacci fibers, multilayer composite fibers, and $n=2$ fibers [20]. After giving an overview of the fibers from [20] in “Hierarchy of symmetry fibers in GRN” section, we study expression of groups of genes predicted to be coexpressed in “Synchronized coexpression within gene fibers—experimental validation” section.

The main approximation behind the existence of perfect synchronization in fibers is the ’uniformity assumption’: the assumption that all the parameters defining the input functions (i.e., the Hill function defining the interaction of the TF with the binding site of the target gene) of the genes in the fibers are the same. That is, the Hill input functions, reflecting interactions of TF binding to DNA, as well as binding parameters or mRNA half lives, needs to be the same for all genes in a fiber to synchronize. Thus, our prediction of perfect synchronization in the fiber depends on a idealized model of gene regulation, where the input functions of genes in the fiber are the same. Under these conditions, fibration theory predicts perfect synchronization in the fiber. Of course, in reality, input functions and interaction parameters of different genes will not be exactly the same, and the question becomes of how much loss of synchronization is caused by this symmetry breaking in the input functions and parameters of interactions.

Despite all the likely reasons for the loss of synchronization ignored in our working hypothesis, including differing gene input functions and various levels of gene regulation, we find a measurable degree of synchronization, matching our simplified theory. We conclude that despite quantitative differences between gene input functions, genes within fibers have measurably more synchronization, which justifies our topological analysis in this case. A further possibility, supported by previously measured gene input functions, is that evolutionary pressures may be at work which “streamline” the gene input functions within fibers, thus preventing a stronger symmetry breaking.

## The fibration formalism and synchronization

The main concepts from graph fibration theory for biological networks—isomorphic input trees, fibers, symmetry fibrations, and the base of a network—have been introduced in Morone, Leifer and Makse [20] (for details, see Methods “Gene fibrations” section) and are based on previous developments by Golubitsky and Stewart [25] and Boldi and Vigna [24]. Examples of symmetry fibrations are displayed Figs. 2, 3, 4, 5 and 6. All these concepts will be exemplified below. In the fibration formalism, a network is described as a directed graph. The input tree of a node represents all paths in the network that lead to this node [20] and it exemplified in Fig. 3 further below. An input tree is constructed by considering the node of interest, which forms the root of the tree, that is, the end point of all the paths leading to that node. If a path contains loops, these loops become “unfolded” in the tree representation. Then, every node in a given layer of the tree represents the initial point of a path in the network leading to the root node. The first layer of the input tree contains all the nodes that are connected by a direct arrow to the root nodes (that is, by a path of length 1). The second layer contains nodes with paths of length 2, and so on. Two trees are called isomorphic if they are topologically identical, where the identity of nodes in the tree does not matter. Nodes with isomorphic input trees are considered equivalent and belong to the same fiber, as exemplified further below in Figs. 2, 3, 4, 5 and 6. A symmetry fibration of a graph *G* [20], called a surjective minimal graph fibration in [24, 42, 43], is a transformation $\psi : G \rightarrow B$ that collapses the nodes in each fiber of *G* into a single representative node, thus reducing the network *G* to a graph *B* called its base (see Figs. 2, 3, 4, 5, 6). In this way, a symmetry fibration reduces a network to its most simple form by compressing the redundancies provided by the symmetric genes in the fibers.

In a GRN—with nodes representing genes or gene products – fibration symmetries have important consequences for gene expression dynamics. Theory developed in [24, 25, 42, 43] shows that a coupled-cell network (in this case gene regulatory network), set of admissible ODEs (ODEs that respect the network structure, is discussed further) and balanced coloring (fibers, equivalence is discussed in Methods “Equivalence between fibers of symmetry fibration and minimal balanced coloring” section) are sufficient for the existence of the synchronized solution corresponding to the fibers of the network irregardless of the specific structure of the ODEs. That is, for the network *G* with fibers $f_1, f_2, \ldots f_n$ consisting of genes $i_1, i_2, \ldots i_{\mid f_i \mid }$ for $i \in \{1 \dots n\}$, where $\mid f_i \mid$ is the size of fiber *i* and each node (gene) is represented by the state variable $x_i$ there exists a solution that is synchronous by fiber i.e.

1 $$\begin{aligned} \begin{array}{ccccccc} x_{1_1}(t) &{} = &{} x_{1_2}(t) &{} = &{} \dots &{} = &{} x_{1_{\mid f_1 \mid }}(t), \\ x_{2_1}(t) &{} = &{} x_{2_2}(t) &{} = &{} \dots &{} = &{} x_{2_{\mid f_2 \mid }}(t), \\ &{}&{}&{} \dots &{}&{}&{} \\ x_{n_1}(t) &{} = &{} x_{n_2}(t) &{} = &{} \dots &{} = &{} x_{n_{\mid f_n \mid }}(t). \end{array} \end{aligned}$$

**Fig. 1:**
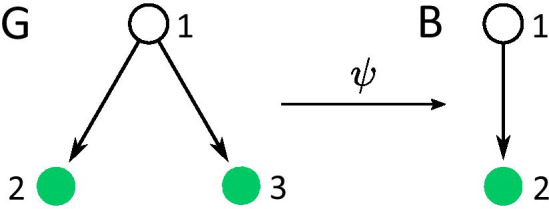
Coupled-cell network of 3 nodes and it’s base. Admissible ODEs corresponding to the network *G* exhibit a synchronous solution $x_2 = x_3$ as a consequence of the existence of fibration $\psi$. This is a simple case of a fibration (corresponding to a trivial regulon) which is shown here only for a didactic purpose. We will show that fibrations describe non-trivial cases below

To gain an intuitive understanding of this concept, let’s consider the simple network in Fig. 1. This network is a trivial example of a fibration which corresponds to a regulon and it is shown here for didactic purposes. This network suffices to introduce the concept of fibration in a simplified setting. We will see that fibrations describe complex non-trivial circuits beyond this trivial case. Fibers will be discussed further in more detail, but for now note that both nodes 2 and 3 only receive one input from node 1 and therefore are input-equivalent. Thus, nodes 2 and 3 belong to the same fiber. Fibration $\psi$ then takes graph *G* to graph *B* by collapsing nodes 2 and 3. Node *i* is associated with the variable $x_i$ and an ODE describing its dynamics. ODEs are admissible [25] if the ODE of node *i* depends on the variables corresponding to the nodes that send the input to node *i* and the variable of the node itself. In this case nodes 2 and 3 receive input from node 1, therefore their ODEs will depend on $x_1$. Admissible ODEs for the network *G* have the form:

2 $$\begin{aligned} \begin{array}{ccl} \dot{x_{1}} &{} = &{} f(x_1), \\ \dot{x_{2}} &{} = &{} g(x_2, x_1), \\ \dot{x_{3}} &{} = &{} g(x_3, x_1). \end{array} \end{aligned}$$

where *f* and *g* are arbitrary functions. Nodes 2 and 3 are modeled with the same function *g* because they belong to the same fiber. $x_i$ in general can represent a *k*-dimensional state vector on $R^k$ phase space. Dimensionality of different nodes can be different as long as nodes in the same fiber have the same dimensionality. Following a similar logic, we write admissible ODEs for the network *B*:

3 $$\begin{aligned} \begin{array}{ccl} \dot{x_{1}} &{} = &{} f(x_1), \\ \dot{x_{2}} &{} = &{} g(x_2, x_1). \end{array} \end{aligned}$$

Take now the solution $(x_1, x_2)$ of the system (3) and plug it in the system (2) such that nodes 2 and 3 are synchronous i.e. $x_2 = x_3$. Then Equation (2) becomes:

4 $$\begin{aligned} \begin{array}{ccl} \dot{x_{1}} &{} = &{} f(x_1), \\ \dot{x_{2}} &{} = &{} g(x_2, x_1), \\ \dot{x_{2}} &{} = &{} g(x_2, x_1). \end{array} \end{aligned}$$

Last two Eqs. of the system (4) are the same equation. Thus, the last equation can be dropped and since $(x_1, x_2)$ is a solution of the system (3), it’s a solution of this system as well. Therefore, the vector $(x_1, x_2, x_2)$ is the solution of the system (2). Hence, the system (2) has a solution that is synchronous by fiber as a consequence of the existence of fibration $\psi$. Note, functions *f* and *g* are arbitrary and $x_i$ can be *k*-dimensional, which allows for simulations of very complex biological processes.

The distinction between the existence of a synchronized solution and synchronization (which means that the synchronous solution is stable) is important. For instance, prediction of time-synchronized expression activity is only possible if (at least local) stability is verified. Depending on the type of coupling and parameter values synchronous solution can be stable or unstable. Additionally, stable solutions can have different size of the basin of attraction. Numerical solutions of the dynamical evolution of the fibers studied here and performed in [22] indicate that for the particular type of interaction fibers found in genetic networks are stable and have a very big basin of attraction. Analytical investigation of the stability and attractiveness of these solutions is out of the scope of this paper, but it can be done using the approach introduced by Pecora, Sorrentino and collaborators [44–47].

Since genes in fibers are synchronized and therefore redundant from the point of view of the dynamics, the symmetry fibration reduces the network to a smaller number of nodes, while preserving the dynamical state of the network. As discussed, this statement relies on a strong uniformity assumption about Hill input functions, which will be scrutinized in subsequent sections.

To summarize, fibration is a mapping between graphs that collapses fibers. Fibers are nodes that have isomorphic input trees and can be collapsed together by map (fibration) with these properties. Fibrations symmetries is the term that we use to describe the symmetries that we are using. The fibers represent the functional set of synchronized genes in the GRN. For the GRNs of *E. coli* and *B. subtilis* and other species, we have previously organized the different types of observed fibers into a hierarchy, reflecting the different complexity of the topological features of their input trees [20, 22]. This hierarchy identifies a broad range of fibers: 91 in *E. coli* (see [20] for the full list of fibers and Table 2 for the distribution of the 85 fiber sizes between 2 and 24) and 216 in *B. subtilis* (203 fibers of sizes 2 to 24 are considered and their size distributed is shown in the Table 1). Despite their various topologies, the resulting fibers can be concisely classified with just two numbers, called ’fiber numbers’ $|n, \ell \rangle$, which specify the number of loops in the fiber, *n*, and of external regulators of the fiber $\ell$ [20].

The fibrations of GRNs can be computed by algorithms to find ’minimal balanced coloring’ available in the literature [20, 48]. Equivalence between fibers and minimal balanced coloring is discussed in Methods “Equivalence between fibers of symmetry fibration and minimal balanced coloring” section and a more detailed description of the algorithm is given in Methods “Algorithm for balanced coloring to identify fibers” section. Code to find fibers is available at https://github.com/ianleifer/fibrationSymmetries.

Mathematical work on fibrations [24, 25] has been concerned with the existence of synchronized solutions, but in some cases realization of the synchronized solution is quite unprobable. We can imagine a trivial case of a network with just 2 nodes with no edges. These two nodes can be synchronized since synchronous solution exists for the two nodes, yet, these solutions may not be most probable (small basin of attraction) since they require some ad-hoc set of initial conditions. In terms of the mathematical definition of symmetry fibrations, the existence of these synchronous solutions is consistent. However, in the real system we obviously do not require these two nodes to belong to the same fiber. We solve this problem in the following way. We first find all strongly connected components with no input (including single nodes with no input) and assign them to different fibers. In this way we avoid finding these stray solutions.

## Gene regulatory networks of *E. coli* and *B. subtilis*

In this paper, we first review the fibers found in the GRNs of *E. coli* and *B. subtilis* studied in [20, 22] and then organize this rich set of fibers into a well-defined hierarchy. At the most simple and trivial level in this hierarchy, we find the known structures of operons and regulons that trivially lead to synchronization. The hierarchy then builds up to more complex architectures as the fiber numbers $|n, \ell \rangle$, describing topological features of the fibers such as *n* loops and $\ell$ regulators, increase, and progresses to fibers like the autoregulation loops, feed-forward fibers, Fibonacci fibers, $n=2$ fibers and multilayer composite fibers.

**Table 1:** Distribution of the fiber sizes of *B. subtilis* for sizes between 2 and 24

Size	Count	Size	Count	Size	Count	Size	Count
2	65	7	8	12	5	21	2
3	40	8	2	13	1	22	1
4	27	9	4	15	2	23	1
5	16	10	6	16	2	24	1
6	12	11	6	18	2		

The studied networks are constructed from data in RegulonDB for *E. coli* [49] and Subtiwiki [50] for *B. subtilis*, well curated resources for gene annotations, regulation, and function in the two bacteria. From the TF-gene bipartite networks we construct a GRN with directed weighted links. Nodes of these networks represent genes and edges are interactions between source gene producing TF (source) that binds to the DNA sequence upstream to the other gene (target). Edges are considered as three different types according to their function: activation, repression, and dual.

The regulons are the first (trivial) members in this hierarchy with a structure represented by no loops and $\ell$ regulators: $|n=0,\ell \rangle$. Next, the scheme classifies non-trivial fibers by $|n=1,\ell \rangle$ as feed-forward fibers (FFF) and autorregulation loops (AR), and $|n=1,\ell \rangle$ as binary tree fibers. The list is completed with more complex fibers with non-integers fiber numbers called Fibonacci fibers with $|\phi _d, \ell \rangle$, where $\phi _d$ is the generalized golden ratio, and composite multilayer fibers as combinations of the fibers above. We have shown in [22] that this set of fibers arises as a constructive procedure that mimics a growth procedure by recursively iterating a constructive process that expands the existence of all fibers in the network. We elaborate on this hierarchy in the rest of this section. In “Synchronized coexpression within gene fibers—experimental validation” section, we shall test the biological significance of these fibers by testing the prediction of synchronization inside the fibers.

## Hierarchy of symmetry fibers in GRN

An important concept in this work is the distinction between coexpression resulting from trivially sharing the same operon and regulon versus synchronization induced by more complex symmetry fibration. That is, the difference between coregulation by fiber (coexpression resulting from shared input trees which take into account extended paths in the network) versus coregulation by a single input of a regulon. Both of them lead to coexpression, but the former is more complex than the latter, which is considered trivial synchronization, while the fiber synchronization is not. We ellaborate on these distinction in the next two subsections.

### Operons

The trivial building blocks leading to synchronization are operons and regulons. Operons are gene arrangements ubiquitous in bacteria [1]: genes in an operon have a common promoter and are not transcribed into individual mRNAs, but as transcription units, yielding a single mRNA strand containing several contiguous genes. Depending on the locations of promoters and terminators, the transcription units can also be overlapping. The expression of genes in an operon will be automatically synchronized since they are translated together. In the case of multi-promoter operons, we can group the genes into minimal transcription units, each being controlled by the same combination of promoters. The genes in such a minimal units should be precisely coexpressed (operons with several terminators can be subdivided similarly). In our analysis we take the operon as a single node in the GRN. When two or more TFs belong to the operon, we leave one TF associated with the operon and separate the remaining TF from the operon.

### Regulons

**Fig. 2:**
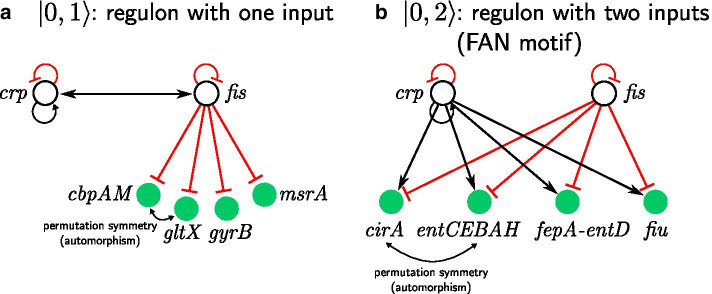
Trivial circuits leading to synchronization: Regulons with co-regulation. **a** Genes *cbpAM, gltX, gyrB* and *msrA* are controlled by the same TF (*fis*). Fiber numbers describing this circuit are $|n = 0, \ell = 1 \rangle$ since there are no loops and fiber has 1 regulator. Gene activity can synchronize, because any two nodes can be permuted without the change in the network under the $S_4$ symmetry group. *fis* won’t be synchronized with *cbpAM, gltX, gyrB, msrA*, because it can’t be permuted with any of the genes without changing network. **b** Regulon circuit consisting of genes *clrA, fiu* and operons *entCEBAH, fepA-entD* controlled by two regulators *crp* and *fis* also synchronizes, because symmetry group $S_4$ is conserved irregardless of the number of the regulators. In this case fiber has two regulators and no loops and therefore is characterized by fiber numbers $|n = 0, \ell = 2 \rangle$

Beyond operons, the next trivial network structure that can synchronize genes is the regulon, defined as the set of genes regulated by a single TF. Figure 2a shows an example, the regulon of the transcription factor Fis in *E. coli*. The regulon contains four units: the operon *cbpAM* and the genes *gltX, gyrB, msrA*. Gene *fis*, is also in turn regulated by Crp. Crp and Fis are two master regulators involved in a myriad of functions, the most important is carbon utilization. This regulon is an example of a first form of simple symmetry, assuming that the genes have no other regulators: a simple permutation symmetry (called automorphism) of the regulated genes, for instance *cbpAM* $\leftrightarrow$ *gltx*, or any permutations between the four genes (Fig. 2a). This symmetry is described by a symmetry group called the symmetric group $S_n$, which consists of all permutations of *n* nodes, in this case $S_4$. This symmetry implies that all the genes in the regulon are synchronized by trivial coregulation of *fis*.

When the genes in a regulon are also under the control of other TFs, then there is also a chance that the regulons would preserve symmetries. For instance, the single-regulon circuit controlled by *fis* (Fig. 2a) can be augmented by a second regulator as in Fig. 2b. The same symmetric group $S_4$ describes the symmetry between the genes *clrA, fiu* and operons *entCEBAH, fepA-entD* since all of them can be permutated. These genes are synchronous, but not with the regulators *crp*, *fis*.

Following the nomenclature for fibers developed in [20, 22], we characterize these circuits by fiber numbers $|n = 0, \ell \rangle$ since they have no loops, $n=0$, inside the fiber and $\ell$ external regulators.

It is interesting to compare the circuits found by fibrations with the most commonly used network motifs. In the network motif nomenclature of Alon et al., the $|0,2 \rangle$ fiber shown in Fig. 2b is called FAN motif [15], while the $|0,1\rangle$ fiber depicted in Fig. 2a is called a star motif. We will see next that in order to synchronize the regulator with its regulon, and extra autoregulation loop is required to induce a fibration symmetry, leading to the first form of non-trivial fiber beyond the regulons and simple symmetric groups $S_n$, as we show next.

### The autoregulation (AR) loop and regulated genes

**Fig. 3:**
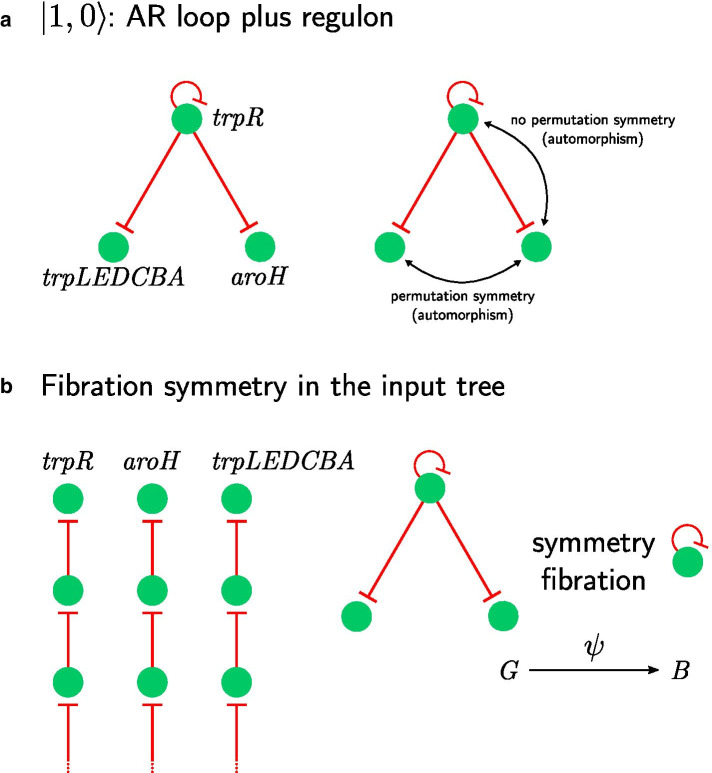
Non-trivial circuits leading to synchronization: AR loop with regulon. **a** Genes *aroH* and *trpLEDCBA* can be permuted under $S_2$ symmetry group, while *trpR* can’t be permuted with them without changing the network. **b** *trpR* receives input only from itself, therefore its input tree is an infinite chain. *aroH* and *trpLEDCBA* receive input from *trpR*, that in turn receives input from itself turning these input trees into chains too. Therefore, input trees of all 3 genes are isomorphic to each other. Thus, *aroH*, *trpLEDCBA* and *trpR* belong to the same fiber and can synchronize their activity. Circuit has one loop and no external regulators, therefore it is classified as $|n = 1, \ell = 0 \rangle$

The first non-trivial form of synchronization in the hierarchy of symmetry fibrations is found when a TF regulates its own expression, forming an autoregulation (AR) loop, and further regulates other genes. This is exemplified in Fig. 3a. In *E. coli*, such a circuit is found in the biosynthesis of tryptophan, which is regulated by TrpR [53], which represses itself, the gene *aroH* (2-Dehydro-3-deoxyphosphoheptonate aldolase), and the *trpLEDCBA* operon, which codes for the enzymes of the tryptophan biosynthesis pathway. This circuit is turned on by the presence of intracellular level of L-tryptophan [54]. When tryptophan is in the cell the TF binds and turns off the genes in the operon.

While the AR loop at *trpR* does not affect the automorphisms formed by the regulated genes (i.e., the regulated genes are invariant under permutations of the symmetric group S$_2$), the AR loop introduces the fibration symmetry between *trpR*, *trpLEDCBA*, and *aroH*. This symmetry is not captured by a simple permutation of the nodes. For instance, permuting the operon *trpLEDCBA* with *aroH* preserves the adjacency, but permuting *trpR* with either the operon or *aroH* does not preserve adjacency. Therefore *trpR* does not belong to the symmetry group formed by *trpLEDCBA* and *aroH*. Still, as we show next, *trpR* is synchronized with *trpLEDCBA* and *aroH* by the symmetry fibration of the input trees. We call this circuit $|1,0\rangle$ since it contains an AR loop and no external regulators.

### The feed-forward fiber

When an AR circuit with its regulon fiber is regulated by an external TF, it becomes a $|1, 1 \rangle$ fiber. This is a prominent circuit in bacteria; we call it the feed-forward fiber (FFF) [20]. The FFF resembles the feed-forward loop (FFL) network motif introduced in [19], except for the additional AR at the intermediate TF. This crucial addition transforms a FFL into a FFF composed of three genes into a synchronized fiber [22]. This type of building block is abundant in *E. coli* and *B. subtilis* GRNs [22].

**Fig. 4:**
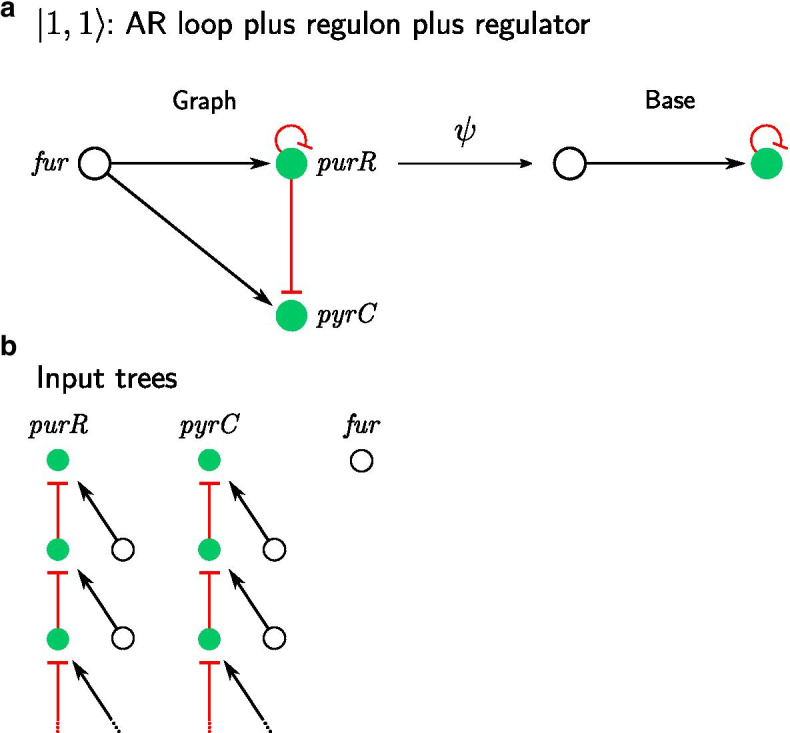
Non-trivial circuits leading to synchronization: FFF (AR loop with regulon and external regulator). **a** *purR* and its target gene *pyrC* regulated by *fur* form a FFF. FFF has one loop and one external regulator and therefore is classified as $|n = 1, \ell = 1 \rangle$. *purR* and *pyrC* belong to the same fiber (will be shown in **b**) and therefore are “collapsed” under fibration $\psi$, while *fur* is left untouched. **b** *purR* receives an input from itself creating an infinite chain and regulator *fur*, that doesn’t have any inputs. Therefore, infinite chain with additional input on each layer represents an input tree of *purR*. Similarly, *pyrC* receives input from *purR* that leads to the infinite chain and *fur* that creates an additional input. *fur* doesn’t receive any inputs and therefore has an input tree of height 0. Input trees of *purR* and *pyrC* are isomorphic, therefore *purR* and *pyrC* belong to the same fiber and synchronize their activity

An example of an FFF is observed in the purine biosynthesis circuit in *E. coli*, shown in in Fig. 4a. It is composed of the repressor TF *purR* and its target gene *pyrC*, both regulated by the master regulator *fur*. The input trees of the genes in this FFF are shown in Fig. 4a. We see that *pyrC* and *purR* receive the same inputs from both *fur* and *purR*. On the first layer of the input tree, we find that *purR* is an autoregulator and also regulates the gene. The second level of the input tree contains exactly the same genes, and so on. This forms an input tree of infinitely many layers since there is a loop in the fiber at *purR*.

### Multilayer composite fiber

Synchronization of the fiber is defined by the isomorphism between input trees of the nodes in the fiber. Consider the input tree of the FFF building block in Fig. 4. First layer for both *purR* and *pyrC* contains the regulator (*fur*) and the green node (*purR*). Therefore, the second layer not only has the same topology, but it has the same nodes. Hence, there is no way to break the isomorphic topology after this layer, because inputs of the same nodes are considered. Therefore, in the case of the FFF building blocks and all the other building blocks considered so far one layer of the input tree or the input set alone is enough to detect synchronization. The next level of complexity in the hierarchy of fibers are circuits where the synchronization depends on deeper input layers of synchronized genes, and longer loops of information. This increase in complexity of the circuits is seen in multilayer composite fibers and Fibonacci fibers which we treat next.

**Fig. 5:**
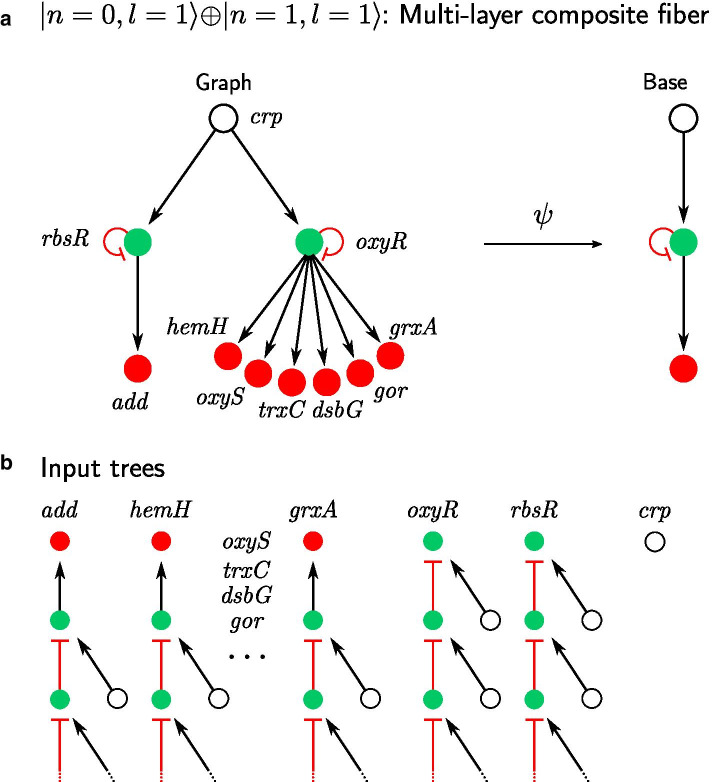
Non-trivial circuits leading to synchronization: Multi-layer composite fiber. **a** Circuit consists of two layers of fibers: *add, dsbG, gor, grxA, hemH, oxyS, trxC* classified with $|n = 0, \ell = 1 \rangle$ and *rbsR, oxyR* classified with $|n = 1, \ell = 1 \rangle$, therefore forming a multi-layer composite fiber $|n = 0, \ell = 1 \rangle \oplus |n = 1, \ell = 1 \rangle$. Fibration $\psi$ of this circuit “collapses” both fibers and leaves the regulator untouched. **b** Genes in the red fiber receive one input from the gene in the green fiber, which in turn receives an input from itself and the regulator. Therefore, input trees of genes in the red fiber resemble the sum of an input tree of $|n = 0, \ell = 1 \rangle$, followed by the input tree of $|n = 1, \ell = 1 \rangle$. Input trees of the green fiber are those of the FFF. Regulator node has no inputs. Thus, multi-layer composite has two non-trivial fibers that can synchronize their activity. Note, gene *add* is separated from the rest of the red fiber by two steps, therefore allowing for a long range synchronization in the network

Figure 5a shows an example of a multilayer composite fiber in *E. coli* whose main regulator is *crp*. In this case, *crp* is the inducer of a composite fiber, composed of *oxyR* and *rbsR* and responsible for further downstream regulation of several carbon utilization subsystems of genes.

The topology of the input trees of this fiber is isomorphic to the one of FFF $|1, 1 \rangle$, despite the fact that the building block has a very different topology than the FFF shown in Fig. 4a. This first layer of genes regulates via *oxyR* and *rbsR* a second fiber composed of genes *add, dsbG, gor, grxA, hemH, oxyS, trxC*. If the branch corresponding to *rbsR* is disregarded, the building block of the fiber of genes *dsbG, gor, grxA, hemH, oxyS, trxC* is classified as a single layer $|0, 1 \rangle$. Thus, the building block corresponding to the entire fiber in Fig. 5a in red is a double layer composite that we denote: $|add - oxyS \rangle = |0, 1 \rangle \oplus |1, 1 \rangle$.

Notice that gene *add* is two edges apart from the rest of its own fiber genes, thus achieving synchronization at a distance of two in the network.

Multilayer fibers are the predominant way for distant synchronization, indicating the higher level of complexity in these composite circuits.

### Fibonacci fibers and the strongly connected component of the network

**Fig. 6:**
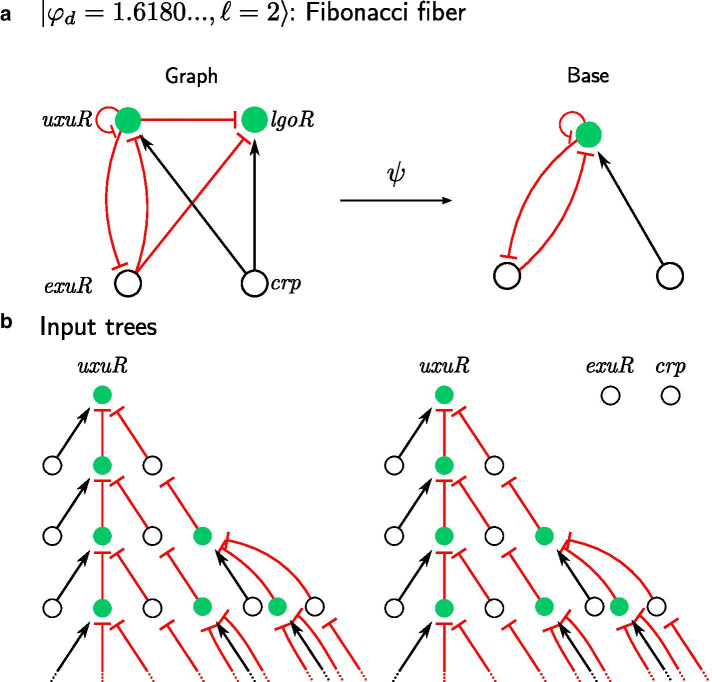
Non-trivial circuits leading to synchronization: Fibonacci fiber (FF). **a** FF circuit is the FFF circuit with the additional edge from the fiber back to the regulator. In this example *uxuR* sends back to *exuR*, creating an extra loop in the circuit. Extra edge won’t change the fiber, therefore fibration will stay the same. **b** However, extra loop changes an input tree of fiber nodes. *uxuR* receives from itself and *exuR*, which in turn receives from *uxuR*, which creates an input tree with layer sizes following Fibonacci sequence. Branching ratio then defines the first fiber number and this FF is classified as $|\varphi _d = 1.6180\ldots , \ell = 2 \rangle$. Note, node *lgoR* receives an input from *exuR* and then from *uxuR*, which means that even if there was no link from *uxuR* to *lgoR*, information would still be passed along through the regulator. This is another way how networks can process the information

The next stage in our hierarchy is the Fibonacci fiber (FF) shown in Fig. 6a [20, 22]. The FF shows a higher level of complexity in the paths that regulate the fiber. To understand the FF, we use the concept of strongly connected component, SCC [20]. In general, a fiber may receive information from the entire network through its input tree. When the fiber is not connected to a SCC, then information is processed only inside the fiber. This was the case with all fibers described so far and characterized by integer fiber numbers $n=0, 1, 2$.

However, if a fiber is connected to a SCC and sends back information to its own regulators through the SCC, the level of complexity in the fiber topology increases. In previous examples, the input trees were infinite due to self-loops, here the input tree becomes infinite due to longer loops in the SCC of the network, i.e., information cycles through a longer loop returning back to the fiber. The input tree of the fiber contains longer loops in the information cycles arriving to the root gene. These loops introduce extra terms in the sequence layers in the input tree leading to Fibonacci sequences in the number of paths, (see [20] for details). There are an infinite number of possibilities for these cycles to appear in the network. We find three types of Fibonacci fibers in the GRN of *E. coli* in our previous work [20], one of which is shown in Fig. 6a. Eukaryotes like yeast and humans present a much richer variety of Fibonaccis as shown in [22].

### $|n=2, \ell \rangle$: binary tree fiber

The last type of building block in the hierarchy of fibers in bacteria is characterized by two AR loops, leading to a symmetric input tree. This procedure can be iterated to any number of loops, but in the studied bacterial networks we did not find any fiber with $n>2$, suggesting a practical limit in complexity in these organisms.

## Synchronized coexpression within gene fibers—experimental validation

We saw that gene fibrations, in theory, can lead to synchronization. To see whether this prediction takes place in reality, we now consider the gene fibers uncovered in bacteria and confront the predicted coexpression structures with experimental transcriptome data (for details see Methods  “Gene expression data” section). We use the gene expression compilations from Ecomics [60] (for *E. coli*) and SubtiWiki [50] (for *B. subtilis*). The Ecomics portal collects microarray and RNA-seq experiments from different strain and sources including NCBI Gene Expression Omnibus (GEO) public database [61] and ArrayExpress [62]. The data is also compiled at the Colombos web portal [63]. We choose Ecomics over Colombos because Ecomics provides absolute expression levels. Datasets for gene expression like Colombos [63] do not provide the absolute expression levels but the fold change from a wild-type to a perturbation state such as a mutation. Measuring the fold change does not allow to test the synchronization in fibers since the prediction of the theory refers to unperturbed states in the wild type. Thus, we base our analysis on the wild-type networks. Expression data from mutant strains were not taken into account, since mutations lead to breaking of symmetries. Theoretical predictions for the response to mutations will be studied elsewhere.

Subtiwiki is a comprehensive knowledge database for bacterium *B. subtilis* containing expression, pathways, interactions and regulation data in the wild-type strain across different experimental conditions. A test for gene synchronization in fibers has been performed in our previous study in [20] by looking at specific experimental conditions where the genes have been activated. Below, we test the existence of fibers in a larger context with and without activated conditions and test the statistical significance of these correlations, as assessed by *p* values.

We assess synchronization in gene expression data using Pearson coefficient of correlation. To find the Pearson coefficient of correlation between gene expression profiles of genes *i* and *j* we use:

5 $$\begin{aligned} C(i,j)= \dfrac{1}{T} \sum _{t=1}^{T} \Big (\frac{x_{i,t}-\mu _i}{\sigma _i}\Big )\Big (\frac{x_{j,t}-\mu _j}{\sigma _j}\Big ), \end{aligned}$$

where *T* is the number of conditions, $x_{i,t}$ is the expression value of gene *i* for condition *t*, and $\mu _i$ and $\sigma _i$ are the respective mean and standard deviation of expression values of gene *i* for all conditions.

**Fig. 7:**
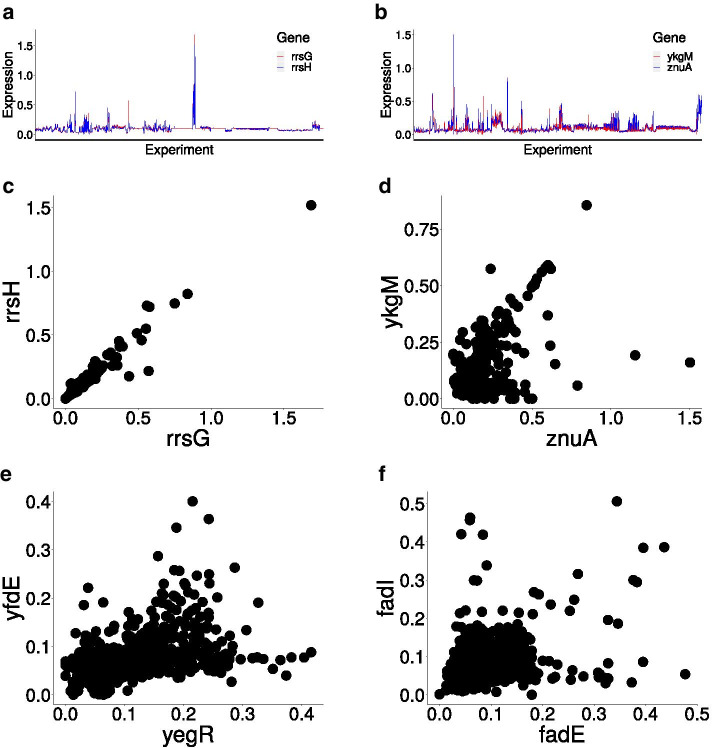
Similarity in gene expression data for selected pairs of genes belonging to the same fiber. Gene co-expression is demonstrated on the data from all experiments in the Ecomics database. We pick best examples out of 85 fibers obtained in *E. coli*. **a, b** Gene expression of pairs of genes in *rrsH, rrsG* and *ykgM, znuA* for 1575 experimental conditions from Ecomics. It’s easy to see that data is highly correlated. **c** Gene expression of *rrsH* (Position in genome: 223771–> 225312) vs gene expression of *rrsG* (Position in genome: 2729616 <– 2731157), correlation = 0.98, **d** Gene expression of *ykgM* (312514 <– 312777) vs gene expression of *znuA* (1941651 <– 1942583), correlation = 0.58, **e** Gene expression of *yfdE* (2488023 <– 2489168) vs gene expression of *yegR* (2167989<– 2168306), correlation = 0.49, **f** Gene expression of *fadI* (240859 <– 243303) vs gene expression of *fadE* (2459159 <– 2460469), correlation = 0.49

We start by considering expression of few pairs of nodes that are predicted to be synchronized by fibration theory in *E. coli*. Figure 7 shows the gene expression correlations between four different gene pairs, each from one fiber, in the form of scatter plots. Each plot can be quantified by a single Pearson correlation value. Figure 7c depicts the expression of *rrsH* vs *rrsG* which form a fiber, and are predicted to be synchronized. Each point in the plot represents a single experiments with a particular growth condition for the bacterium as obtained from the Ecomics dataset. The observed Pearson correlation value is 0.98 which indicates a strong synchronization in the activity of the genes across the experimental conditions. These genes are located far away in the genome and, as it can be seen from the scatter plot, their expression is highly correlated. Figure 7d–f shows another few scatter plots of gene expression in *E.coli*. For instance, fig. 7f shows the correlation between *fadI* and *fadE* which also form a fiber with a correlation coefficient 0.49. These highly correlated examples are picked only for illustrative purpose, but not all fibers synchronize so well, and as it can be seen from Fig. 7 there is a lot of noise.

In the following sections we assess whether the observed correlations are significantly large within the predicted fibers by assessing gene correlations in the entire data set, within and between gene fibers. We report two kinds of correlations: (a) correlations computed from the entire data set, that is, using all the experimental conditions appearing in Ecomic for *E. coli* without filtering, irrespective of whether the genes are being expressed in the particular conditions or not, and (b) correlations obtained after filtering for “active experimental conditions”, specifically chosen for each set of genes in the fibers. This second approach has been used in our previous study in [20] and it is similar to the filtering method employed by the Colombos database at [63]. Then, we scrutinize the statement that fibrations predict larger mean correlation (i.e. gain of synchronization) in the fibers of *E.coli*. Again, we first give significant results without filtering and we continue by applying the variation of the filtering method used by Colombos [63] and showing significance and results of that method.

### Correlations within and between gene fibers

In this subsection we give an overview over the correlations between all genes in the dataset, and their relation to gene fibers. We start by grouping the genes in the predicted fibers (85 in total in *E. coli*) and then using all the experimental conditions in Ecomics (1575 conditions) we calculate the correlation matrix between and within genes in fibers to test for synchronization. Later we will filter these conditions to those where the genes are active.

**Fig. 8:**
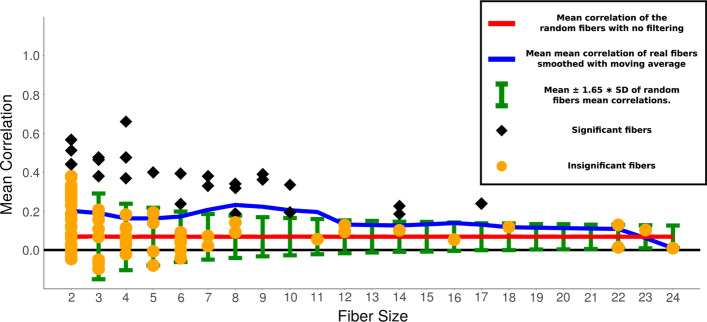
Mean correlation within the fibers, computed without filtering versus sizes of gene fibers (number of nodes). Black and orange dots—mean correlation of 85 fibers of size < 25. Shape shows significance: black diamond—significant, orange circle—insignificant. Blue—mean of mean correlation of real fibers in black (smoothed with moving average). Green error bars—$mean \pm 1.65 * SD$ of random fibers mean correlations. 1.65 is chosen, because 0.05 values of the normal distribution with $\mu =0$ and $\sigma =1$ are above 1.65, therefore 1.65 corresponds to the *p* value of 0.05. Red—mean correlation of the random fibers

In order to quantify the synchronization in the fibers we consider the statistics of mean correlations inside the fiber, where mean correlation is the mean of all off-diagonal terms in the correlation matrix. To assess the statistical significance of the correlations we compare the fiber mean correlations with mean correlations of random sets of nodes of the same size. We assume that mean correlations of random blocks of a given size are distributed normally. We define mean and standard deviation of the random set by finding mean correlation of 100,000 random sets of fibers of size ranging from 2 to 24 genes (as found in *E. coli*) using all the conditions of expression data. Then we find mean and standard deviation of this 100,000 random sample. Summary of this analysis is shown in Fig. 8. In general we find that the mean correlation for the genes inside the fiber is relatively low, with all fibers with mean correlation below 0.5. We will see below that this is due to the fact that in many experimental conditions from Ecomic the fibers are not activated. However, it is also clear from the data that there is an increase in correlation inside the fibers (blue curve in Fig. 8) as compared with the mean correlation in the random sets (red curve in Fig. 8), but how significant is this increase?

In order to quantify the statistical significance of the increase in correlations in fibers, we study the probability that random sets of genes of size *n* have the mean that is higher or equal than the mean correlation of fibers of the same size *n*. We then calculate the *p* value of the measured distribution belonging to the random distribution.

The sampling distribution of a normal distribution of size *m* has the mean distributed normally with mean ($\mu _m=\mu$) and standard deviation $\sigma _m = \frac{\sigma }{\sqrt{m}}$, where $\mu$ and $\sigma$ are mean and standard deviation of the original distribution [64]. For a normal distribution with known mean and standard deviation, we find a z-score that corresponds to our measurement, i.e. to the mean of mean correlations of fibers $\mu _{real}$ as $Z = \frac{\mu _{real} - \mu _m}{\sigma _m}$ and the corresponding *p* value. Table 2 shows the summary of these measurements. We find that 68 out of 85 fibers are significant, which indicates that for most of the fibers the mean correlation is significantly higher than random. Therefore fibers significantly predict the gain of synchronization even in this unfiltered dataset. However the typical mean correlation is low enough (below 0.5) to consider this result of high significance.

**Table 2:** Significance of the increase in correlation obtained using method with no filtering

Fiber size	Mean of mean correlations ($\mu _{real}$)	Mean of random mean correlations ($\mu _m$)	Standard deviation of random mean correlations ($\sigma _m$)	Number of blocks (*m*)	*p* value
2	0.20	0.07	0.20	24	**0**
3	0.17	0.07	0.13	11	**0.01**
4	0.20	0.07	0.10	10	**0**
5	0.14	0.07	0.09	6	**0.03**
6	0.10	0.07	0.08	8	0.11
7	0.20	0.07	0.07	4	**0**
8	0.22	0.07	0.07	5	**0**
9	0.38	0.07	0.06	2	**0**
10	0.26	0.07	0.06	2	**0**
11	0.06	0.07	0.05	1	0.60
12	0.11	0.07	0.05	2	0.12
14	0.17	0.07	0.05	3	**0**
16	0.05	0.07	0.04	1	0.63
17	0.24	0.07	0.04	1	**0**
18	0.12	0.07	0.04	1	0.11
22	0.07	0.07	0.04	2	0.45
23	0.10	0.07	0.04	1	0.17
24	0.01	0.07	0.03	1	0.95

*P* values < 0.05 in bold. 68/85 are significant. Random sample consists of 100,000 fibers

### Inverse Coefficient of Variation to filter out conditions with non-activated genes

**Table 3:** Significance of the increase in correlation obtained using ICV

Fiber size	Mean of mean correlations ($\mu _{real}$)	Mean of random mean correlations ($\mu _m$)	Standard deviation of random mean correlations ($\sigma _m$)	Number of blocks (*m*)	*p* value
2	1	0.99	0.08	24	0.32
3	0.81	0.78	0.17	11	0.25
4	0.78	0.58	0.22	10	**0**
5	0.53	0.46	0.22	6	0.24
6	0.44	0.38	0.20	8	0.20
7	0.50	0.32	0.19	4	**0.03**
8	0.51	0.28	0.18	5	**0**
9	0.77	0.25	0.17	2	**0**
10	0.55	0.22	0.16	2	**0**
11	0.25	0.20	0.14	1	0.37
12	0.20	0.18	0.14	2	0.44
14	0.28	0.15	0.12	3	**0.03**
16	0.07	0.14	0.11	1	0.74
17	0.50	0.12	0.10	1	**0**
18	0.19	0.11	0.09	1	0.21
22	0.10	0.10	0.08	2	0.43
23	0.23	0.09	0.07	1	**0.03**
24	0.02	0.09	0.07	1	0.84

*P* values < 0.05 in bold. 28/85 are significant. Random sample consists of 100,000 fibers

**Fig. 9:**
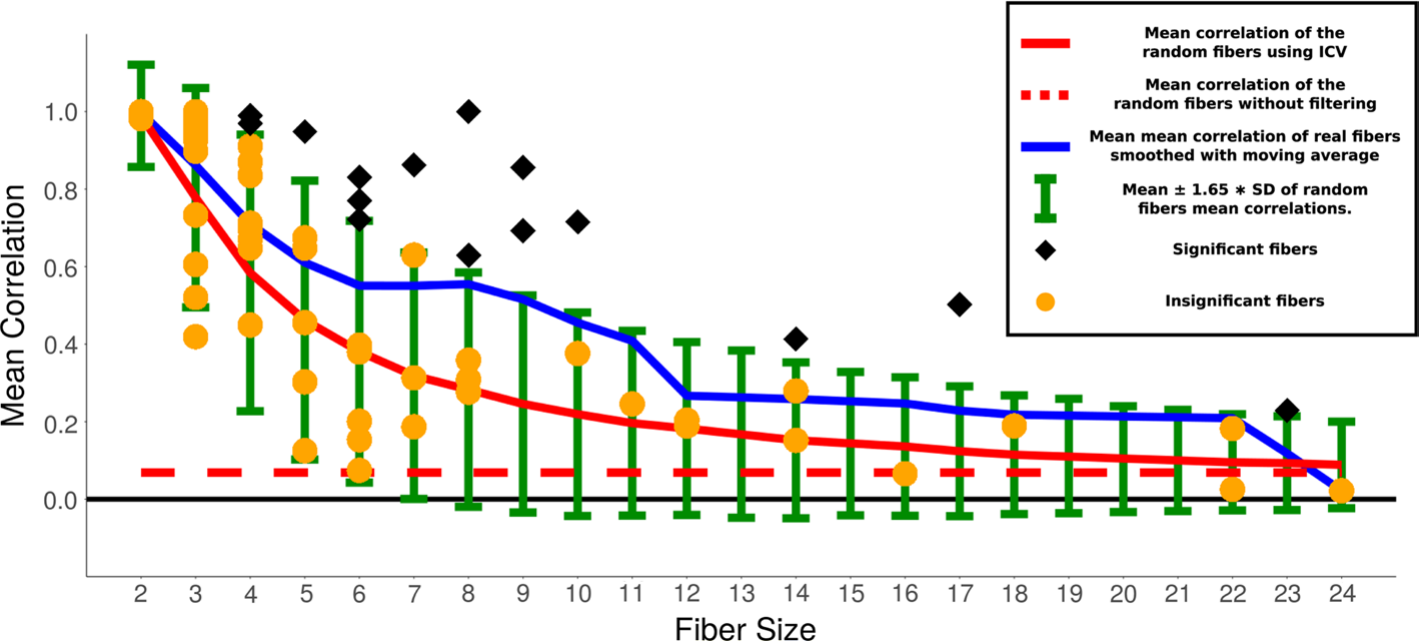
Mean correlation of fibers calculated using the filtering method of ICV vs number of nodes in the fiber. Black and orange dots—mean correlation of 85 fibers of size < 25. Shape shows significance: black diamond—significant, orange circle—insignificant. Blue—mean of mean correlation of real fibers in black (smoothed with moving average). Green error bars—$mean \pm 1.65 * SD$ of random fibers mean correlations. 1.65 corresponds to the *p* value of 0.05 as explained in Fig. 8. Red solid line—mean correlation of random fibers found using ICV. Red dashed line - mean correlation of the random fibers without filtering

The results so far refer to gene correlations across the entire data set, including all experimental samples. However, in our theoretical predictions we assume that correlations in a fiber should only exist when the fiber is “active”, that is, in a subset of “active” experimental conditions, which is specific to this fiber. Since we don’t have any other information about these active conditions, next, we determine a set of active conditions, for each single fiber, by a heuristic criterion, based on the ICV (Inverse Coefficient of Variation, see Methods). It is important to note that this filtering for high ICV values is, basically, also a filtering for conditions in which the genes tend to be correlated: i.e. our filtering for active conditions will induce correlations. To see which of these correlations represent “real” correlations and not just statistical artifacts, we rely again on our permutation test.

Indeed, cells can adapt to varying growth conditions by sensing extracellular cues, using intracellular effector molecules to modulate transcription factor activities. This activation can happen directly or via intermediate signaling pathways such as the two-component systems (TCS) in bacteria. Therefore, different genes will not be expressed all the time, but will only be expressed under specific external experimental growth conditions. We consider that a gene is either active (expression much larger than noise range) or inactive (expression in the noise range). Furthermore, the activity of all TFs are modulated by effectors (ligands and metabolites). In our approach, these additional regulations are ignored and requiere a detailed consideration of the metabolic networks that is coupled to the GRN. Such coupling will be studied in a forthcoming paper. In the present analysis we consider that these effectors activate and deactivate the fiber circuits identified by fibrations and are determined by the internal metabolism of the cell and the external growth conditions where fibers are activated.

A specific example of activation mediated through a known effector is the cAMP activation of *crp*. When the genes are not significantly expressed or expressed below the noise level, the corresponding activity in expression is expected to be random noise. When the genes are active or significantly expressed for a given statistical test of significance, the genes can be coexpressed by showing large correlations in their expression levels or they cannot be coexpressed, by showing zero or near noise level correlations in their expression levels over time.

When the expression correlations between genes are computed from the entire set of conditions as done in the previous section, the noise in the conditions where genes are inactivated distorts the results. That is, using the inactivated states to compute the correlations, there will be noise, which makes it hard to detect correlations, leading to the need to filter the conditions.

Thus, to test coexpression patterns in a given set of genes, we first find the conditions under which this set of genes is active, ie, over-expressed under a given statistical test. Then we assess, just for these conditions, the coexpression between our genes. Being inactivated together by external effectors, we filter out for experimental growth condition where the fibers are activated and present correlations over a given determined threshold of noise. We note that activation of genes does not imply correlations per se. Thus, there are two different stages in the analysis that are subject to different statistical tests of their significance as we explain below.

For this study we use Ecomics since it provides the data in the wild-type (WT) conditions, rather by providing the data using the fold-change that compares a mutation or perturbation to the WT. Using Ecomics [60], we obtain the set of experimental conditions where the particular genes in a given fiber have been significantly expressed. For this task we follow standard gene expression analysis similar to the one developed in colombos.net and Ref. [63] for the expression levels in *E. coli* to first identify the set of growth condition where the genes in a given fiber are significantly expressed respect to random noise and then test the synchronization through correlations in gene’s activity using these conditions. We then repeat the scheme using the conditions in Subtiwiki for *B. subtilis* [50].

To choose our gene sets to test for synchronization, we first consider the building block of the fiber. We test the synchronization in the fiber of the building block and the lack of synchronization between the fiber and its external regulators. We then consider the cross-correlations between fibers.

For a given set of genes in a fiber, we find the experimental conditions for which the genes have been significantly expressed by comparing the expression samples over different biological conditions. To filter conditions where the genes are expressed we use the Inverse Coefficient of Variation (ICV) similar to the one applied by Colombos [63]. We consider the genes in the fiber and obtain their expression levels for all conditions. Then we calculate the ICV for all conditions using the method explained in “Selecting relevant experimental data based on the inverse coefficient of variation” section.

After selecting the conditions for expression according to the relevant ICV, we use the expression data for the selected experimental conditions and we find the Pearson correlation coefficient of correlation between gene expression profiles of genes *i* and *j*. For genes that are in the same fiber, we calculate the correlation matrix averaging over the experimental conditions of the fiber using the ICV method explained above. To compute correlations between genes belonging to different fibers, we consider the correlation function calculated over the union of conditions used for two fibers.

The above framework yields a correlation matrix for a fiber and its regulators. We apply the method to each fiber in the networks of *E. coli* and *B. subtilis* to test the prediction that genes in the fiber are more correlated with genes in the fiber, than with genes outside the fiber.

To deal with the noise generated by the inactivated states of the genes, we filter the conditions based on the ICV [63]. ICV allows us to consider only conditions where mean expression is few standard deviations higher than 0, that is $\mu _{expression} > n * \sigma _{expression}$, where n is an arbitrary number (see Methods). In other words, we consider conditions where the fiber is activated. This filtering could create a bias towards increased correlation, so the question arises of how significant results obtained using this method are.

The analysis of gene correlations with ICV filtering was performed as follows. We considered each fiber, determined the active conditions for this fiber, and computed the intra-fiber correlations over this set of conditions. To compute gene correlations across two different fibers, we considered the previously determined active conditions for both fibers and computed the correlations over these conditions.

Figure 9 shows the summary of the results similar to the one presented before in Fig. 8 for the method with no filtering by comparing the significance of the correlations in the fibers with a null model of 100,000 random set of genes with sizes from 2 to 24. First, we observe that mean correlation in the random set approached 1 when the size of the set approaches 2. This implies that for fibers with two genes, the filtering method is not significance, i.e., any random set of genes will show high correlations after filtering the conditions with ICV. However, the average of the within-fiber mean correlation for a given size of fiber as a function of the size of the fiber (red curve in Fig. 9) slowly decays towards the red dashed line, which means that the correlation bias created by ICV disappears for bigger sizes of the fiber. Second, we can see a clear increase of mean correlation (blue line being higher than red) similar to the one we observed in the method with no filtering. This implies that we again see the increase in correlation within the fiber using the method of ICV. Significance of this increase can be studied observing Table 3. We see that 28 out of 85 fibers are significant. The proportion of the fibers that are significant using the ICV method is lower than without filtering, which is mostly happening due to the fact that the increase in the correlation for the smaller sizes is less significant.

Having studied the correlations across all genes in fibers with filtering and no filtering of conditions, we conclude that there is a clear pattern of increase of correlation between genes in the fiber. The method with no filtering shows that small fibers detect the significant gain of synchronization, while the method with filtering shows the gain of synchronization in bigger fibers. It’s important to note that there are fibers that are significant and highly correlated individually as it can be seen from Figs. 8 and 9, but what we observe here is a consolidative effect of gain of synchronization, although with extra fiber-fiber correlations which are not directly related to the fiber structure but may indicate activation of different fibers under same conditions. We shall now describe some particular cases of fibers with biological functionalities.

**Fig. 10:**
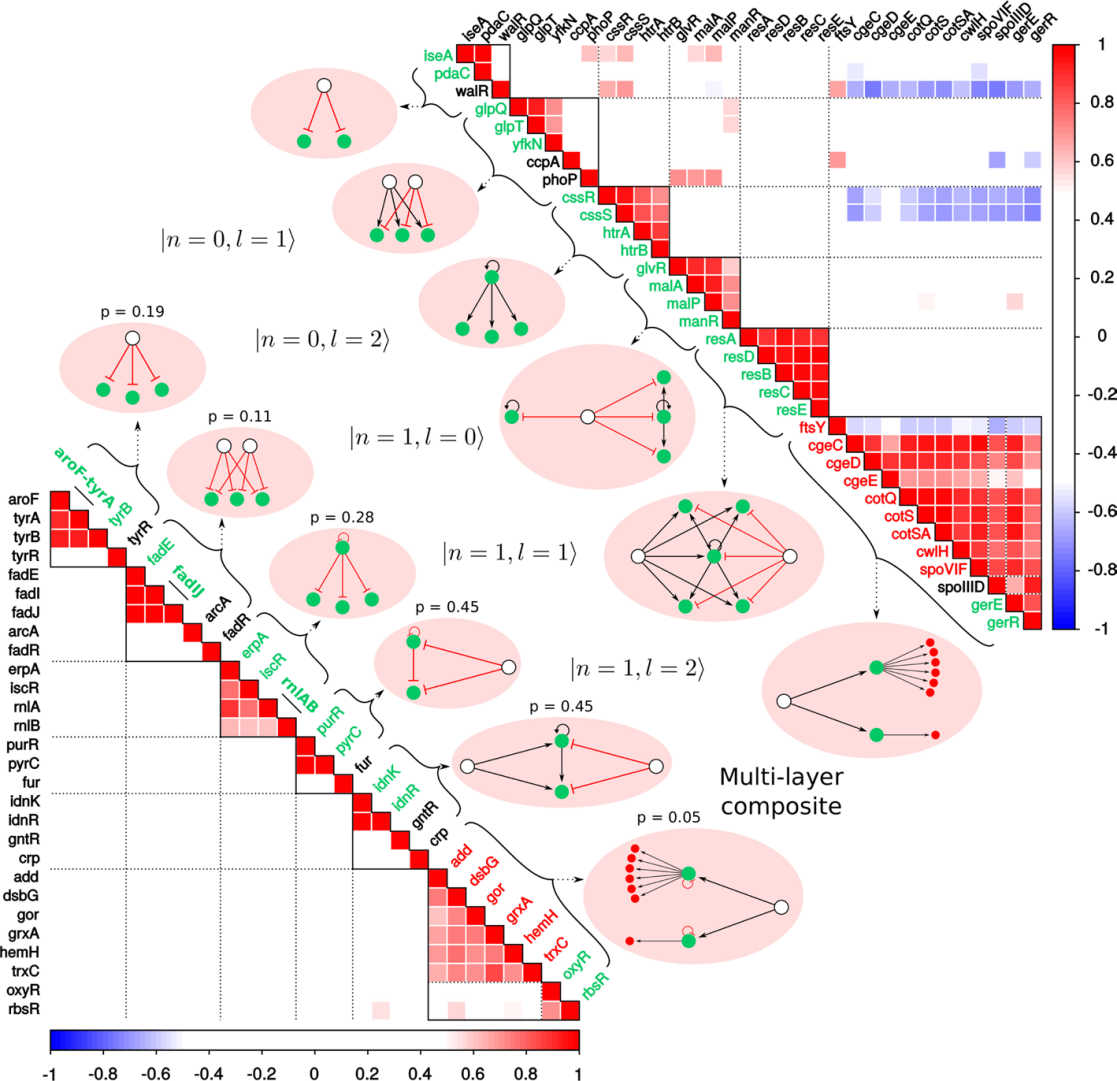
Fiber Class Examples. We display the six different fiber classes with their genetic circuit and correlation matrix. *Genetic circuits:* A graphical representation of the genes and their regulators interactions. Edges: Black—Activation, Red—Repression. Nodes: Green and red—Fibers, White—Regulators. *Correlation graphs:* Correlation between Fiber genes (green and red font) and regulators (black font). Operons are shown with lines along the correlation matrix diagonal. Black lines in the correlation matrix enclose fibers, black dotted lines show cross-correlations between fibers and inside multi-layered fibers. Genes inside fibers are correlated and are not correlated with regulators and different fibers. Note, observed correlations have high *p* values using ICV method. This happens due to the fact that the displayed fibers are small and, as mentioned before, small fibers are have high *p* values with method with no filtering. Observed correlations can guide future research in finding missing transcriptional regulations. For example, self-regulation loop on *spoIIID* could explain the correlation inside multi-layered fiber in bacillus

### Fiber synchronization in the hierarchy of fibers in *E. coli* and *B. subtilis* at the network level

So far we have tested coexpression within fibers separately, selecting for each fiber the conditions under which the genes in this fiber are activated. Next, we consider cross-correlation between a selected set of fibers well studied in the literature with concomitant high activation levels since many conditions have been set in experiments to study their behavior, to check the validity of our results. A synchronization across fibers might indicate that our gene fibers are not correct, maybe because of missing edges in the reconstructed network.

To calculate the correlations inside fibers we again use the conditions where each fiber has been overexpressed as given by the ICV. In general, these sets of conditions need not overlap for two different fibers. When calculating the off-diagonal correlation between different fibers we consider the union of the conditions of both fibers to calculate the correlation matrix.

Figure 10 shows the expression correlation matrix for genes in a number of circuits in *E. coli* and *B. subtilis*, following the hierarchy of fibers explained in “Hierarchy of symmetry fibers in GRN” section. We study the following fibers: $|0, \ell \rangle$ with $\ell =1, 2$, followed by three examples of Chain Fibers $|1, \ell \rangle$ with $\ell =0, 1, 2$ and by a case of a multilayer composite, present in both species. Looking at the multilayered fiber in* B. subtilis* in Fig. 10 we observe synchronization of the fiber and it’s regulators. Since this multi-layered building block in bacillus is fully synchronized, we predict that there should be a AR on the regulator SpoIIID that will turn this in a $|1, 0 \rangle$ fiber in order to explain this synchronization. Finding these missing links is a useful byproduct of the existence of symmetries, which can be done systematically to find and annotate new regulatory interactions.

The activity of the genes in operons are reported individually in Ecomics, so we use the activity of the individual genes (genes in one operon are marked in the plots). Synchronization within operons is a trivial finding, and the test of fiber synchronization is done by comparing the activity of any gene in the operon with the genes outside the operon. Moreover, the fiber predicts no synchronization between any gene in the operon and the external regulator.

We note the lack of synchronization between the fiber genes *purR-pyrC* and its regulator *fur* as predicted by fibrations. This is despite the fact that the fiber is the regulon of *fur*, that is, direct regulation does not lead to synchronization. As predicted, genes are highly coexpressed within fibers, but not significantly correlated with the regulator genes.

Observed correlations largely confirm the synchronization within fibers. However, there are some interesting exceptions. For instance, the genes *cssR* and *cssS* in *B. subtilis* present large anticorrelations with a fiber (the one containing the gene *cgeC*). This unexpected anti-correlation may indicate extra transcriptional regulations between these fibers. These type of correlations can be used to guide in the search for missing regulation edges, which are ubiquitous in genetic network reconstructions.

### Coexpression of regulators of alternative carbon source utilization

**Fig. 11:**
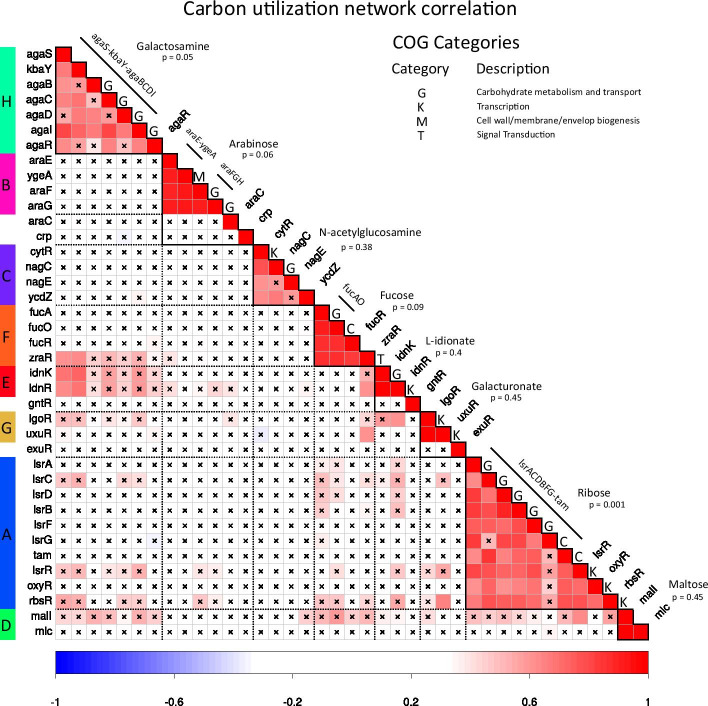
Carbon utilization circuit: correlation matrix. Correlation matrix of the fiber building blocks involved in the carbon utilization system. Colored rectangles $A, B \dots H$ on the left code gene names that will be used in expression matrix plot Fig. 12 and structure vs function plot Fig. 13. Operons are shown with lines along the correlation matrix diagonal. Black crosses show correlation entries below 0.6 to compare low cross-correlation with high correlation inside fibers. COG categories are obtained using UniProt database [65]. Function of each block (Galactosamine, Arabinose, etc.) is defined by the type of it’s regulator obtained from RegulonDB [49]

**Fig. 12:**
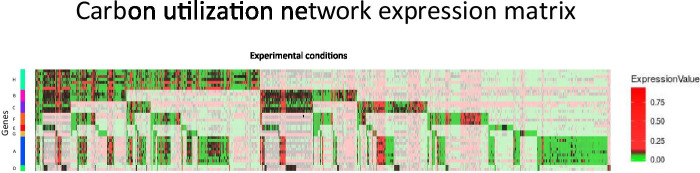
Carbon utilization circuit: expression profile. Expression profile of 39 genes involved in the carbon utilization system over 1575 experimental conditions. Gene names correspond to the building blocks $A, B \dots H$ defined in Fig. 11. Conditions in white are filtered out using the method of ICV described in “Selecting relevant experimental data based on the inverse coefficient of variation” section and the rest of the conditions are used to calculate the correlation represented in Fig. 11

**Fig. 13:**
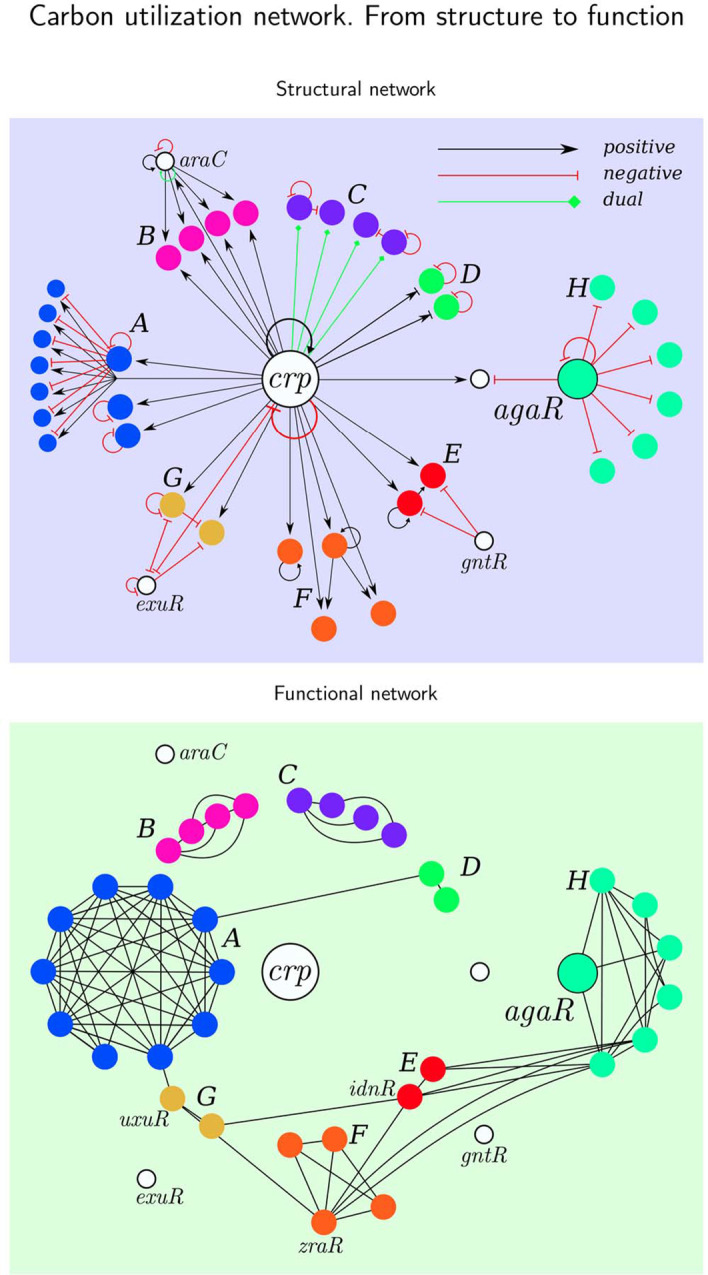
Carbon utilization circuit: structural network vs functional network. Top part shows topology of the transcriptional regulation of the carbon utilization system. Bottom part shows the functional network of the carbon utilization system based on the Pearson correlation from Fig. 11. Correlation *C*(*i*, *j*) is thresholded at $C(i,j) >0.6$ to produce a functional network

The underlying assumption in the functional annotation of genes is that the genes that are highly connected in modules have a functional relation among them; an assumption that is usually called ’guilty by association’ [2, 40]. We now scrutinize this gene annotation method in light of the existence of fibers.

For this purpose we take a closer look at a number of fibers involved in the well studied functional module regulated by the master regulator *crp* involved in the regulation of carbon source catabolism, a well-characterized gene regulatory system. We will also study how the fibers are integrated into a larger network. It has previously been found that this circuit contains many types of network motifs, including feed-forward loop (FFLs), FANs and others [15, 38]. We will see that these network motifs are not related to the synchronized fibers.

To build the carbon utilization network we select those building blocks in which the TF (that generates the input tree) belongs to the alternative carbon sources functional category [66] that catabolizes sugars in the absence of the main source of sugar intake that is glucose. This includes the catabolism of a number of sugars: maltose, ribose, galactosamine, fucose, N-acetylglucosamine, L-idonate, and galacturonate. Correlation matrix of the regulation network is shown in Fig. 11. Gene expression profile corresponding to this network demonstrating filtering explained in Section VII G is shown in Fig. 12. Topology of the regulation network is shown in Fig. 13.

Even thought genes involved in carbon utilization have a related common biological function, that is the assimilation of alternative carbon sources, these genes may not be expressed all together. Indeed, each unit of this group must be activated in the presence of the effector involved in the regulation. Different building blocks are activated under different nutrient intakes of sugars like the presence of arabinose, maltose or ribose. Most of these systems are additionally regulated by the master regulator *crp*.

We start the analysis with the TF AgaR that catabolizes Galactosamine that is regulated by *crp*, this TF regulates the fiber *agaS-kbaY-agaBCDI*. AgaR has several binding sites on the downstream region of AgaS. The binding sites repress the promoter agaSp. Also agarR is only regulated by AgaR with two binding sites on the downstream region of gene *agaR* near promoter agaRp. From literature [38] it is known that in the arabinose system, *araJ* and *araE* code for low-affinity transporters, while the *araFGH* operon codes for a high-affinity transporter, these transport systems differ in other properties in addition to their affinity for their substrate, that is, they have different physiological properties. The *araFGH* operon is subject to strong catabolic repression, that is, it is not expressed if glucose is present in the medium. On the other hand, the low-affinity transporter *AraE* works at moderate arabinose concentrations. The expression of this transporter favors the entry of the substrate and the expression of the enzymes that are going to metabolize.

We found that the genes in the alternative carbon utilization system show measurable coexpression within fibers, just as predicted (see Fig. 11). The coexpression in the fibers (e.g., malI, mlc) is captured by the fibration symmetry. This result shows also that the share of input functions among genes, like in a regulon/operon does not necessarily lead to synchronization.

We have also found fibers that contain genes with different input functions like the ones in ribose circuit, yet, they still have measurably high co-expression. They are also correlated by function, since NagE is a N-acetylglucosamine PTS permease, while YcdZ is a putative transmembrane protein that have been predicted to interact with several sugar PTS permeases (Uniprot). Thus, fibers do not only hint at synchronization, but also at putative functional relations.

We also observe many network motifs in the carbon network as it was found previously [15, 17, 66–69]. For instance the genes *gntR*, *idnR*, *idnK* form a FFL as shown in Fig. 13. However these network motifs are not necessarily related to the functional fibers of synchronized blocks. Finally, the structure-function relation predicted by fibrations is shown in Fig. 13. This relation cannot be predicted by building blocks built from network motifs [17] nor modularity analysis based on community detection methods [40, 70].

## Summary and outlook

Their potential for implementing controllable gene coexpression and for facilitating gene rearrangements make gene fibers an interesting tool of analysis not only for studying existing GRNs but also for synthetic biology. Our results show that gene fibers can capture measurable gain of synchronization in gene expression in two well-reconstructed genetic networks, those of *E. coli* and *B. subtilis*. With this experimental confirmation of our symmetry hypothesis, fibration symmetry seems to be a plausible starting point for a broader theory of gene synchronization. Such a theory would start with the description of exact symmetries and would then proceed with perturbative schemes, allowing for heterogeneities, based on controlled loop expansions on the theory [71]. The overall goal would be a predictive framework for gene synchronization, including an assessment of the effects of mutations. In the present study, we focused completely on wild-type strains. It would be interesting to study knockout experiments, where fibrations might predict a loss of synchronization in comparison to the wild-type predictions.

## Methods

### Network construction

The gene regulatory network of *E. coli* was obtained from the operon dataset from RegulonDB [49] with additional filtering. An operon starts with a promoter, but can also have internal promoters and terminators. In a study in *E. coli*, about 45% of all genes were found to be single genes, about 20% were in traditional operons with one promoter (plus 7% for operons with several terminators), and about 20% were in operons with internal promoters (plus 8% for operons with internal terminators) [72]. In our networks, we consider each operon as a single node, unless an operon contains several TF, in which case, the TFs are considered individually separately, but the genes in the operons that do not express a TF, like for instance enzymes, are considered as a single node together with one of the TF in the operon. For instance, the operon *gadAXW* in *E. coli* is considered as the operon *gadAX* which includes one TF, and the other TF, GadW. For detailed description of filtering process in *E. coli* see [20] (SI Chapter III).

The *B. subtilis* gene regulatory network was obtained from SubtiWiki [50] with additional filtering. All sigma factor genes were removed from the network. Additionally all types of link like “positive_regulation”, “transcription_activation” and “transcriptional_activation” were assigned as “activation” and “anti-activation”, “auto-repression”, “negative_autoregulation”, “transcription_repression”, “autorepression” and “negative_regulation” as “repression”.

### Gene fibrations

When two nodes in a directed graph have isomorphic input trees, the nodes are symmetric and synchronize their activity even if they are not in the orbit of an automosphism. In this case, the synchronized nodes are said to belong to the same fiber [20, 22, 24, 73, 74]. The symmetry fibration is then a transformation that collapses nodes in a fiber into a single node called the base and thus reduces the circuit to its most simple form. The “orbits” of synchronized genes in an automorphism are now called the nodes in the fiber produced by the symmetry fibration.

Groups of nodes that share fibration symmetry are called fibers. Nodes that have isomorphic input tree belong to the same fibers of minimal fibrations (further referred as fibrations for simplicity) [24]. For example the input trees of all the genes in the tryptophan circuit are isomorphic. They consist of a infinite chain as shown in Fig. 3b, since this circuit contains one single loop and no external regulators that do not belong to the fiber. Thus, we characterized it by the fiber numbers $|1, 0\rangle$. The symmetry fibration is a transformation that reduces this circuit by collapsing all nodes in the fiber to one, called the base. This is only possible since all genes in a fiber are redundant in a dynamical state.

### Equivalence between fibers of symmetry fibration and minimal balanced coloring

Equivalence between fibers of symmetry (surjective minimal) fibration and minimal balanced coloring (or coarsest equitable partition) is formally proven in Chapter 4 in [74]. In particular, Theorem 4.7 states that maximal balanced equivalence relation is equivalent to the isomorphism relation between input trees of the infinite depth. That is, minimal balanced coloring induced by the maximal balanced equivalence relation is equivalent to having classes of nodes with isomorphic input trees, which correspond to the fibers of the symmetry fibration. Rigorous proof requires a fairly involved mathematical analysis [74], so we will only give a brief idea here. Consider graph *G* with fibers $f_1, f_2, \ldots f_n$ and balanced coloring $C=\{c_1, c_2, \ldots c_m\}$.

1. Let there be two nodes $n \in f_i$ and $m \in f_j$ that belong to different fibers and have the same color $c_k$. Since *n* and *m* are of the same color, they will have isomorphic input trees. Therefore, there exists a fibration $\varphi$ that can collapse $f_i$ and $f_j$. Hence, $\psi$ is not minimal, which contradicts the assumption.

2. Let there be a fiber $f_i$ that breaks into two colors $c_j$ and $c_k$. Since *C* is minimal, nodes of the different colors will have input trees that are not isomorphic. Therefore, there is a node $n \in c_j$ and a node $m \in c_k$ input trees of which are not isomorphic. Therefore, *n* and *m* can’t belong to the same fiber, as required.

Consequently, there can’t be any two nodes that belong to the same fiber, but have different colors and the opposite. Ergo, fibers of symmetry fibration are equivalent to minimal balanced coloring.

### Algorithm for balanced coloring to identify fibers

Several algorithms can be used to find fibers in networks [49, 74, 75]. All available algorithms are based on finding ’balanced equivalence’ relations in the network, see [25] for details. Current algorithms are based on the algorithm introduced in 1982 by Cardon and Crochemore [75]. In [20, 22] we have used the version developed by Kamei and Cock [49]. A detailed explanation of this algorithm is given in [20, 22]. In a recent review article we further discuss a fast algorithm that is scalable to large system sizes in [76]. The code of the algorithm in R can be accessed at https://github.com/ianleifer/fibrationSymmetries.

### Gene expression data

Expression data for *E. coli* were obtained from Ecomics [61] (Multi-Omics Compendium for *E. coli*). Ecomics contains microarray and RNA-seq experiments gathered from NCBI Gene Expression Omnibus (GEO) [62], for several* E.coli* strains in 1575 different experimental growth conditions for 4096 genes. We only used data from WT strains. The advantage of Ecomics datasets compared with others compilations of expression experiments like Colombos [64] is that they provide absolute expression levels instead of fold-changes. Expression data for *B. subtilis* were obtained from SubtiWiki [51]. Subtwiki contains data from the GSE27219 experiment in GEO that has 104 experimental conditions for genes in wild type *B. subtilis*.

Using these data for a global analysis would be difficult since they stem from different platforms used by the different experimental groups. Thus, raw data on gene expression among different experiments from different labs is pre-processed by the curators of Ecomics and SubtiWiki to produce normalized expression levels across platforms and experiments by using noise reduction and bias correction normalized data across different platforms. For our analysis, we selected data from wild-type strains only (selecting WT conditions in ’strain’, ’medium’ and ’stress’) to ensure the behavior of genes on standard growth conditions without genotype modification from gene knockouts. The ’perturbation’ conditions in the Ecomics dataset referring to mutants strains were not taken into account, and we use always the same *E. coli* strain.

### Data and code availability

The datasets used in this study are available at Refs. [50] and [51] and code for fiber finder used in this study can be downloaded at https://github.com/MakseLab. Expression data from* E. coli* and* B. subtilis* along with codes reproducing co-expression analysis are available at https://github.com/makselab/geneCoexpressionFibration.

### Selecting relevant experimental data based on the inverse coefficient of variation

To select experimental samples in which a gene set of interest is active, ie, significantly expressed above random noise level, we used the Inverse Coefficient of Variation (ICV) as a criterion similar to the approach used by Colombos [64]. We consider the genes in the fiber and obtain the expression levels for all conditions for the genes. Then we calculate ICV for all conditions using the following equation as is done in Colombos (details on the expression analysis can be found at Ref. [64] and at https://doi.org/10.1371/journal.pone.0020938.s001):

6 $$\begin{aligned} ICV_t=\dfrac{{\mu _t}}{\sigma _t}, \end{aligned}$$

where $\mu _t$ is the average expression level of the chosen genes of the fiber in the condition *t* and $\sigma _t$ is the standard deviation. Following [64], we select conditions with $ICV_t> <ICV_t>$, i.e., where the average expression levels in the particular condition *t* are higher than certain threshold that is given by the average ICV for all conditions of the fiber. This score reflects the fact that, in a relevant condition, the genes show an increment on their expression above the individual variations caused by random noise. ICV is a measure of scattering of the data. The more scattered the data is compared with it’s mean, the less is the value of ICV.

We calculate the *p* value of condition *t* for the fiber genes using z-score of the ICV coefficient for the selected condition using

7 $$\begin{aligned} z_t = \frac{ICV_t-\mu _{ICV}}{\sigma _{ICV}}, \end{aligned}$$

where

8 $$\begin{aligned} \mu _{ICV}= & {} <ICV_t>, \end{aligned}$$

9 $$\begin{aligned} \sigma _{ICV}= & {} \sqrt{<ICV_t^2>- <ICV_t>^2}. \end{aligned}$$

## Data Availability

R package to reproduce the building blocks in* E. coli* and* B. subtilis* is available at https://​github.​com/​ianle​ifer/​fibra​tionS​ymmet​ries and https://​github.​com/​makse​lab. Expression data from* E. coli* and* B. subtilis* along with codes reproducing co-expression analysis are available at https://github.com/makselab/geneCoexpressionFibration.
